# Deficient Neutrophil Extracellular Trap Formation in Patients Undergoing Bone Marrow Transplantation

**DOI:** 10.3389/fimmu.2016.00250

**Published:** 2016-06-27

**Authors:** Jared W. Glenn, Mark J. Cody, Meghann P. McManus, Michael A. Pulsipher, Joshua D. Schiffman, Christian Con Yost

**Affiliations:** ^1^Division of Neonatology, Department of Pediatrics, University of Utah School of Medicine, Salt Lake City, UT, USA; ^2^Division of Pediatric Hematology, Oncology, and Bone Marrow Transplant, Department of Pediatrics, University of Utah School of Medicine, Salt Lake City, UT, USA

**Keywords:** bone marrow transplantation, neutrophil, neutrophil extracellular traps, infection, innate immune response

## Abstract

Overwhelming infection causes significant morbidity and mortality among patients treated with bone marrow transplantation (BMT) for primary immune deficiencies, syndromes of bone marrow failure, or cancer. The polymorphonuclear leukocyte (PMN; neutrophil) is the first responder to microbial invasion and acts within the innate immune system to contain and clear infections. PMNs contain, and possibly clear, infections in part by forming neutrophil extracellular traps (NETs). NETs are extensive lattices of extracellular DNA and decondensed chromatin decorated with antimicrobial proteins and degradative enzymes, such as histones, myeloperoxidase, and neutrophil elastase. They trap and contain microbes, including bacteria and fungi, and may directly affect extracellular microbial killing. Whether or not deficient NET formation contributes to the increased risk for overwhelming infection in patients undergoing BMT remains incompletely characterized, especially in the pediatric population. We examined NET formation *in vitro* in PMNs isolated from 24 patients who had undergone BMT for 13 different clinical indications. For these 24 study participants, the median age was 7 years. For 6 of the 24 patients, we examined NET formation by PMNs isolated from serial, peripheral blood samples drawn at three different clinical time points: pre-BMT, pre-engraftment, and post-engraftment. We found decreased NET formation by PMNs isolated from patients prior to BMT and during the pre-engraftment and post-engraftment phases, with decreased NET formation compared with healthy control PMNs detected even out to 199 days after their BMT. This decrease in NET formation after BMT did not result from neutrophil developmental immaturity as we demonstrated that >80% of the PMNs tested using flow cytometry expressed both CD10 and CD16 as markers of terminal differentiation along the neutrophilic lineage. These pilot study results mandate further exploration regarding the mechanisms or factors regulating NET formation by PMNs in patients at risk for overwhelming infection following BMT.

## Introduction

The polymorphonuclear leukocyte (PMN; neutrophil) is the first circulating leukocyte to respond to tissue damage or infection and the primary effector cell of innate immunity and acute inflammation (1, 2). PMNs rapidly infiltrate areas of injury or infection where they participate in wound healing, infection containment, and microbial killing. Disorders associated with a deficiency or impairment of neutrophil number or function, such as neutropenia, chronic granulomatous disease (CGD), or leukocyte adhesion deficiency syndrome, predispose to severe bacterial and fungal infections (3) and are associated with significant morbidity and mortality (1). Novel neutrophil activities continue to be elucidated suggesting that PMNs influence all aspects of immunity (2).

Recently, neutrophils isolated from healthy adult donors were shown to undergo programed cell death distinct from apoptosis and necrosis to form neutrophil extracellular traps (NETs) (4, 5). NETs are extensive lattices of extracellular DNA and decondensed chromatin decorated with antimicrobial proteins and degradative enzymes, such as myeloperoxidase and neutrophil elastase (NE). NETs affect extracellular killing of bacteria and fungi (6–9). We recently identified impaired NET formation as a novel innate immune deficiency in human newborn infants (10). PMNs isolated from the umbilical cord blood of newborn infants, whether born at term or prematurely, demonstrate impaired NET formation and extracellular bacterial killing in the first week of life as compared with PMNs isolated from healthy adults (10, 11). Thus, severe, early onset, neonatal infections may be associated with deficient PMN NET formation leading to impaired extracellular microbial containment and killing.

Pediatric and adult patients undergoing bone marrow transplant (BMT) also exhibit an increased risk for severe infection. A 2006 study found that 85% of cord blood transplant recipients and 69% of hematopoietic stem cell transplant recipients developed at least one severe infection within a 3-year median follow-up time (12). Such infections accounted for 59 and 61% of infection-related mortality for cord blood and peripheral blood stem cell transplant recipients, respectively (12). Posttransplant immune reconstitution is thought to mirror neonatal immune development, and the complicated process of immune system reconstitution continues over 1–2 years (13). However, the risk for microbial infection is greatest in the pre-engraftment period when patients are neutropenic (14). A previous study concluded that an unknown element of neutrophil dysfunction exists in patients after BMT which contributes to the high risk for infection (15). In addition, a recent report looked at NET formation following BMT in 12 adult patients and demonstrated a significant decrease in NET formation compared with healthy adult controls (16). We hypothesized that this increased risk for infection results, in part, from deficient NET formation by neutrophils produced by the nascent, peri-engraftment immune system and tested this hypothesis in a primarily pediatric population. We now show that NET formation by PMNs isolated from pediatric and adult patients before and after BMT exhibit diminished NET formation *in vitro* and that this failure of NET formation is not simply the result of neutrophil immaturity.

## Materials and Methods

### PMN Isolation

Polymorphonuclear leukocytes were isolated from EDTA anticoagulated venous blood of 24 participants either undergoing BMT or after completion of BMT using a research protocol approved by the IRB at the University of Utah. Fourteen of the participants were <18 years old at the time of BMT (Table 1), and the median age for our study participants was 7 years. Eighteen of our study participants only had NET formation assessed after engraftment following BMT. Four of these participants had only qualitative assessment of NET formation using live cell imaging, while the additional 14 of these participants had NET formation assessed both qualitatively and quantitatively. For 6 of the 24 participants, we obtained serial, peripheral blood samples at three different time points: pre-BMT (*n* = 3), pre-engraftment (*n* = 6), and post-engraftment (*n* = 6) (Table 1). For all experiments, we performed assays of NET formation and granulocytic differentiation markers in parallel using PMNs isolated from the peripheral blood of healthy adults as positive controls for NET formation and PMN differentiation (10, 11, 17). In all, we collected peripheral blood from 24 adult donors as controls for the experiments outlined. These adult donors are described as healthy, fasting, medication free, and non-hospitalized, consenting control subjects aged ≥21 years who donate peripheral blood routinely under a University of Utah IRB approved protocol. No attempt was made to match control donors to study participants with regard to age, sex, or ethnicity. PMN suspensions (>96% pure) were prepared by positive immunoselection using anti-CD15-coated microbeads and an auto-MACS cell sorter (Miltenyi Biotec, Inc.) and were resuspended at 2 × 10^6^ cells/mL concentration in serum-free M-199 media warmed at 37°C.

**Table 1:** **Patient characteristics according to age group**.

	Age group (years)			
Characteristics, *n*(%)	Combined	0–10	11–30	(≥31)
***n***	24	15 (62.5%)	6 (25%)	3 (12.5%)
**Gender**				
Female	12 (50%)	9 (60%)	1 (17%)	2 (67%)
Male	12 (50%)	6 (40%)	5 (83%)	1 (33%)
**Primary disease**				
SCID	2 (8%)	2 (13%)	0 (0%)	0 (0%)
Relapsed ALL	6 (25%)	4 (27%)	2 (33%)	0 (0%)
Bilineal leukemia	2 (8%)	2 (13%)	0 (0%)	0 (0%)
Relapsed AML	1 (4%)	1 (7%)	0 (0%)	0 (0%)
Neuroblastoma	3 (13%)	3 (20%)	0 (0%)	0 (0%)
AML	3 (13%)	0 (0%)	2 (33%)	1 (33%)
MDS	1 (4%)	0 (0%)	0 (0%)	1 (33%)
AML/MDS	1 (4%)	1 (7%)	0 (0%)	0 (0%)
ALL	1 (4%)	0 (0%)	1 (17%)	0 (0%)
CLL	1 (4%)	0 (0%)	0 (0%)	1 (33%)
Hodgkins lymphoma	1 (4%)	0 (0%)	1 (17%)	0 (0%)
Anaplastic ependymoma	1 (4%)	1 (7%)	0 (0%)	0 (0%)
Aplastic anemia	1 (4%)	1 (7%)	0 (0%)	0 (0%)
**HLA**				
Matched, unrelated	10 (42%)	4 (27%)	4 (67%)	2 (67%)
PTD of PMN engraftment, mean	20.4	25.3	17.5	16.5
Matched, related	4 (17%)	3 (20%)	0 (0%)	1 (33%)
PTD of PMN engraftment, mean	23	25	–	17
Cord blood	5 (21%)	4 (27%)	1 (17%)	0 (0%)
PTD of PMN engraftment, mean	20.2	16.8	34	–
Autologous	5 (21%)	4 (27%)	1 (17%)	0 (0%)
PTD of PMN engraftment, mean	11	10.8	12	–
**Survival (as of October 2014)**				
Alive	16 (67%)	10 (67%)	3 (50%)	3 (100%)
Dead	8 (33%)	5 (33%)	3 (50%)	0 (0%)

*PTD, posttransplant day*.

### Live Cell Imaging of NET Formation

Qualitative assessment of NET formation was performed, as previously referenced (10, 17). Briefly, participant primary PMNs were incubated with control buffer or stimulated with LPS (100 ng/mL) for 2 h at 37°C in 5% CO_2_/95% air on glass coverslips coated with poly-l-lysine. After stimulation, PMNs were gently washed with PBS and incubated with a mixture of cell permeable (Syto Green, Molecular Probes) and impermeable (Sytox Orange, Molecular Probes) DNA fluorescent dyes. Confocal microscopy was accomplished using a FV300 1X81 Microscope and the FluoView software (Olympus). Both 20× and 60× objectives were used. *Z*-series images were obtained at a step size of 1 μm over a range of 20 μm for each field. The Olympus FluoView software and the Adobe Photoshop CS2 software were used for image processing. Semiquantitative analysis of NET formation was accomplished using the ImageJ analysis software (NIH) and a standardized grid system with rigorous NET quantitation. Statistical comparisons were made *via* one way ANOVA with Tukey’s multiple comparisons *post hoc* testing.

### Quantitation of NET Formation – Supernatant Histone H3 Content

We determined supernatant total histone H3 content as a surrogate for NET formation, as previously referenced (17). After live cell imaging of control and stimulated primary PMNs (2 × 10^6^ cells/mL; LPS 100 ng/mL), the cells were incubated with new media containing DNase (40 U/mL) for 15 min at room temperature to break down and release NETs formed in response to stimulation. The supernatant was gently removed and centrifuged at 420× *g* for 5 min. The cell-free supernatant was then mixed 3:1 with 4× Laemmli buffer prior to Western blotting. We used a polyclonal primary antibody against human histone H3 protein (Cell Signaling Technology) and infrared secondary antibodies (Li-Cor Biosciences). Imaging and densitometry were performed on the Odyssey™ infrared imaging system (Li-Cor Biosciences). Statistical comparisons were made *via* one way ANOVA with *post hoc* testing.

### Assessment of PMN Differentiation

We assessed PMN differentiation through analysis of CD16 and CD10 protein surface expression on PMNs isolated from BMT patients using flow cytometry, as previously described (18). PMNs were isolated by positive immunoselection using anti-CD15-coated microbeads and an auto-MACS cell sorter (Miltenyi Biotec, Inc.) as described (10, 17, 18) and prepared for flow cytometry using FACS lysis buffer (Becton-Dickinson). PMN CD16 and CD10 cell surface protein expression was determined by incubating directly conjugated antibodies against human CD 16 (PE; 10 μL/test) and human CD10- (FITC; 10 μL/test) or isotype-matched control antibodies in the dark at 4°C for 30 min (All antibodies from Becton-Dickinson). FACS analysis was accomplished in the University of Utah Flow Cytometry Core using a Becton-Dickinson FACS Scan Analyzer and Flow-Jo analysis software (Version 9.7.6).

## Results

**Figure 1:**
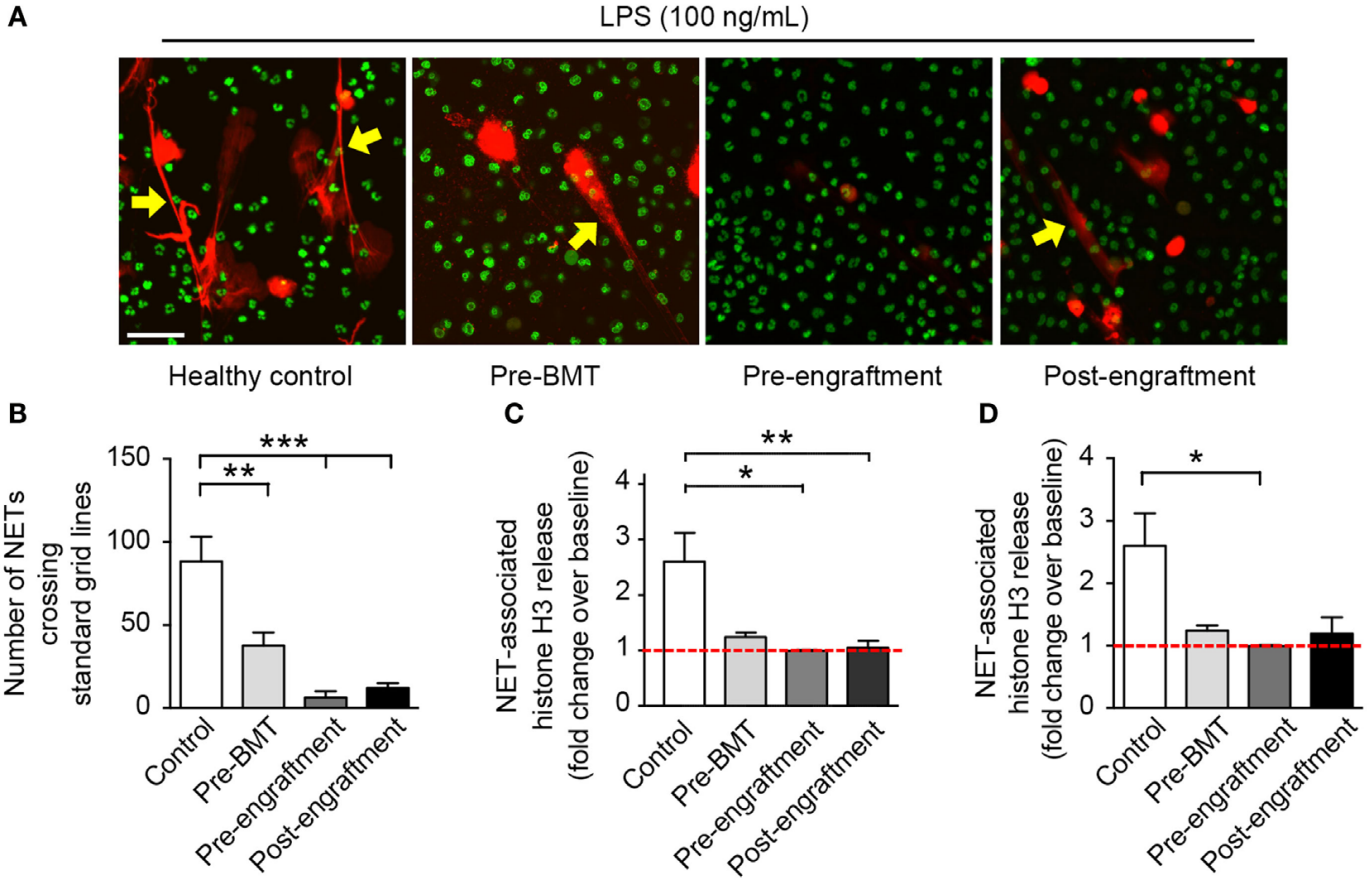
**PMNs isolated from patients at BMT demonstrate decreased NET formation following *in vitro* stimulation with LPS**. We assessed NET formation by LPS-stimulated PMNs isolated from patients undergoing bone marrow transplant (*n* = 3 pre-BMT, *n* = 6 pre-engraftment, *n* = 19 post-engraftment) compared with control LPS-stimulated PMNs isolated from healthy adult donors. PMNs were stimulated with LPS (100 ng/mL; 1 h) and NET formation was determined qualitatively and quantitatively using **(A)** live cell imaging (60× magnification), **(B)** semiquantitative image analysis, and **(C,D)** release of histone H3 (fold change over baseline; mean ± SEM). **(A)** NET-associated, extracellular DNA is shown in red fluorescence (yellow arrows). Nuclear DNA is shown in green fluorescence. **(B)** We analyzed NET formation in a semiquantitative manner using ImageJ analysis software and a standardized grid system for all captured live cell imaging results (20× magnification, *n* > 6 images analyzed per patient group). The *y*-axis depicts the number of times that NETs crossed the standardized grid lines (**p < 0.01 and ***p < 0.001). We employed a one way ANOVA statistical tool with Tukey’s *post hoc* testing. **(C)** NET-associated histone H3 release is shown as fold change over baseline on the *y*-axis (*p < 0.05 and **p < 0.01) compared with baseline (red dashed line), arbitrarily set at 1. We employed a one way ANOVA statistical tool with Tukey’s *post hoc* testing. **(D)** Here, we reanalyze the NET-associated histone H3 release data for only these study participants from whom serial PMN samples were obtained (*n* = 3 pre-BMT, *n* = 6 pre-engraftment, *n* = 6 post-engraftment, and *n* = 11 controls). NET-associated histone H3 release is again shown as for **(C)** *p < 0.05 compared with baseline (red dashed line), arbitrarily set at 1. We employed a one way ANOVA statistical tool with Dunn’s *post hoc* testing.

In this study, we assessed *in vitro* NET formation in response to LPS stimulation by PMNs isolated from 24 BMT patients with varying ages at and indications for BMT (Table 1). In 18 of these BMT patients, we were only able to assess post-engraftment NET formation (posttransplant day range: 14–199). For six BMT patients, however, we assessed peri-engraftment NET formation at the time of BMT, collecting pre-BMT, pre-engraftment, and post-engraftment PMN samples for analysis of NET formation. All six of these patients had received chemotherapy for malignancies prior to pre-BMT conditioning. Chemotherapeutic agents used for these patients included busulfan, carboplatin, etoposide, cyclophosphamide, daunorubicin, vincristine, cytarabine, and mitoxantrone. We did not obtain pre-BMT neutrophil samples from the two patients in this cohort undergoing BMT for severe combined immunodeficiency syndrome and, therefore, did not have pre-BMT chemotherapy. Consistent with a previous report (16), we demonstrated a significant decrease in NET formation by PMNs isolated both pre- and post-engraftment following BMT in response to LPS stimulation as compared with healthy adult control PMNs (Figures 1B–D). Furthermore, using semiquantitative image analysis, we demonstrated a statistically significant decrease in NET formation by LPS-stimulated PMNs isolated from study participants prior to BMT (Figure 1B). While our histone H3 release assay results did not show a correlative statistically significant decrease in NET formation by LPS-stimulated pre-BMT PMNs compared with healthy control PMNs (Figure 1C), an apparent trend toward decreased NET formation was detected (Figure 1C); this correlates with the qualitative and semiquantitative image analysis results (Figures 1A,B).

Of the PMNs isolated post-engraftment from 18 BMT patients, NET formation following LPS stimulation, while detectable, was decreased compared with healthy control PMNs (Figures 1A–C). Furthermore, a subset of patients analyzed showed decreased NET formation despite being 60–199 days removed from their BMT (not shown). This suggests that decreased NET formation may be an unrecognized aspect of PMN dysfunction after BMT that contributes to the prolonged risk for severe infection seen in these patients, which may last up to 3 years (13).

**Figure 2:**
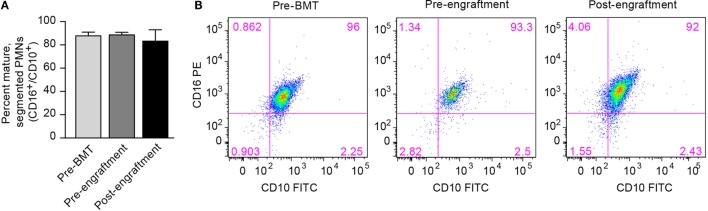
**Fully differentiated and segmented PMNs circulate in cancer patients before and after bone marrow transplantation**. **(A)** We assessed the differentiation of circulating PMNs in three patients undergoing BMT using flow cytometry. Different peripheral blood samples were obtained pre-BMT, after BMT but pre-engraftment, and after engraftment (ANC > 500). Surface expression of CD16 and CD10, two protein markers expressed by fully differentiated PMNs, was determined, and the percentage of PMNs expressing both CD16 and CD10 is shown on the *y*-axis for all three groups of isolated PMNs. No statistically significant differences were noted. **(B)** Representative scatter plot results obtained using flow cytometry are shown with surface CD16 PE expression on the *y*-axis, and surface CD10 FITC expression seen on the *x*-axis. The percentage of PMNs expressing both CD16 and CD10 is shown in the top right corner of each scatter plot.

One possible cause for this prolonged NET deficiency following BMT is that a large proportion of neutrophils isolated pre-engraftment and right after engraftment may be developmental precursors of fully differentiated PMNs. Therefore, we assessed surface expression of the protein markers of PMN differentiation CD16 and CD10 by PMNs isolated from three BMT patients at the pre-BMT, pre-engraftment, and post-engraftment stages. Surface expression of CD16 and CD10 denotes differentiation of granulocytic precursors into fully differentiated, segmented PMNs (19). We found no differences in PMN differentiation between pre-BMT, pre-engraftment, and post-engraftment PMNs, and the PMN preparations from all three groups showed that >80% of the isolated PMNs expressed both CD16 and CD10 (Figure 2).

## Discussion

This brief report demonstrates that NET formation by PMNs isolated from both pediatric and adult patients following BMT is impaired in response to LPS stimulation and that this does not result from incomplete differentiation of the PMNs circulating in these patients. These findings extend the literature as a confirmatory report of decreased NET formation in adult patients following BMT and are the first to show this potential immune deficiency following BMT in pediatric patients. A recent report by Domingo-Gonzalez et al. demonstrated a similar decrease in NET formation by PMNs isolated from BMT patients (16). Their study characterized phorbol-12-mystirate (PMA)-induced NET formation by PMNs isolated from 12 patients undergoing BMT for indications, including various cancers and myelodysplastic disorders. These patients were all adults and the median participant age in their study was 59 years. In contrast, while we did include five adult patients in our study cohort, the median age for our study participants was 7 years. Another difference between our studies was the use of different agonists to induce NET formation. While PMA is a known, non-physiologic PMN stimulatory agonist and does induce NET formation (5), our use of LPS for NET induction more closely approximates the *in vivo* triggering by infectious agents.

Our results are particularly pertinent given the pronounced predisposition of BMT patients, both adult and pediatric, to severe infections, especially in the pre-engraftment period (13, 20). We acknowledge the low sample size for these investigations as a weakness of this pilot study, but note that this study is the largest to date to specifically examine NET formation after BMT in humans and the only one to examine NET formation before human BMT. An additional, potential weakness of this study is the failure to control for age by using age-matched controls in our assays of NET formation. While this represents a potential confounder in this study, we note that age-specific normal values for NET formation in both pediatric and adult populations have not been determined. To date, non-disease specific abnormalities in NET formation have only been characterized in the very young (10, 11) and very old (21). Another study weakness is our inability to examine the effects of autologous versus allogenic bone marrow reconstitution on NET formation following BMT. This very important question will be addressed by planned future studies where a larger number of autologous and allogenic BMT patients can be enrolled. In addition, we also point to the diversity in indications for and times after BMT as strengths of this report suggestive that deficiencies of NET formation in these patients may contribute to their increased risk for severe infection (20). Bacterial and fungal infections continue to cause significant morbidity and mortality in the pretransplantation, pre-engraftment, and post-engraftment periods, which together last through 100 days after BMT (13). While controversy exists regarding the extent of their direct antimicrobial activity, NETs clearly trap bacteria and fungi (22). This effect alone can lead to containment and clearance of an infectious agent, a finding confirmed in a murine model of necrotizing fasciitis with NET inhibition in PAD4 knockout mice (23). Correlative data in the human system also suggest a role for NET formation in clearance of microbes during severe infection. Bianchi et al. reported the use of gene therapy to restore NET formation and to control refractory pulmonary aspergillosis in a patient with CGD (8, 9). These studies and our results suggest a need to examine NET formation prospectively in a larger cohort of BMT patients where the number, types (bacterial, fungal, and parasitic), and severity of infections may be correlated with indications for BMT, pre-BMT chemotherapeutic regimens, and aspects of neutrophil function in addition to NET formation.

Clearly, further studies are warranted to determine the mechanisms or factors leading to this deficit in NET formation after BMT. We have previously shown NET formation to be deficient in undifferentiated HL-60 leukocytes, a cancer cell line arrested in the pro-myelocytic stage of PMN differentiation (17). However, retinoic acid-induced differentiation of HL-60 leukocytes leads to robust NET formation in these leukocytes in response to LPS stimulation (17). These findings led to our initial hypothesis regarding the mechanism for decreased NET formation following BMT. With this study, we have disproven our initial hypothesis that decreased NET formation by PMNs isolated after BMT results from neutrophil developmental immaturity, a finding that might also have been expected given our results in PMNs isolated from newborn infant cord blood (10). In their recently published paper, Domingo-Gonzalez et al. also studied PMNs isolated from a murine model of BMT and demonstrated that increased levels of prostaglandin E_2_ (PGE_2_) in mice following BMT inhibited PMA-induced NET formation (16). This finding is also consistent with the results of Shishikura et al. who showed that PGE_2_ inhibits NET formation by PMA-induced murine and human PMNs through increased production of cyclic AMP (24). Together, these reports elucidate yet another regulatory pathway governing NET formation in human and murine PMNs. Our report, in contrast, focused on PMNs isolated from human BMT patients or healthy controls and examined only one possible reason for decreased NET formation – that of decreased myeloid cell maturity after BMT. Still, future studies in PMNs isolated from BMT patients, both before and after BMT, will need to include interrogation of the many known molecular pathways governing NETosis. Examples of such studies would include an examination of the toll-like receptor signaling pathways; an investigation of BMT effects on intracellular reactive oxygen species generation (5) and autophagy (25), with the BMT effects on the role of NE/myeloperoxidase in triggering NETosis in these patients as well (26); and finally an assessment of PAD4 activity as an enzyme leading to nuclear decondensation, a precursor step toward NETosis (23, 27).

Finally, our findings suggest that PMNs isolated from BMT patients prior to BMT may exhibit a deficit in NET formation and mandate that NET formation be studied in PMNs isolated from other immunodeficiency patients as well as cancer patients in general. If confirmed, NET deficiency by pre-BMT PMNs may be a result of the specific disorders leading up to BMT or from chemotherapy prior to BMT. To date, the effects of cancer chemotherapeutic agents on NET formation by neutrophils have not been extensively studied. Our study examining the role of the mammalian target of rapamycin (mTOR) signaling pathway in the regulation of NET formation did investigate rapamycin, an immunosuppressant and chemotherapeutic agent used in solid organ transplantation and some cancer treatment regimens, as an inhibitor of NET formation. We found that rapamycin inhibited NET formation in an mTOR and hypoxia-inducible factor 1-dependent manner. None of the participants in this report were exposed to rapamycin, and none of the chemotherapeutic agents to which they were exposed have been studied with regard to their effects on NET formation. Clearly, with these findings and the findings of Domingo-Gonzalez et al., further investigation is warranted (16). Alternatively, the question of a circulating inhibitor of NET formation should be considered, with PGE_2_ being a leading candidate. Identification of an endogenous inhibitor of NET formation might prove important given the newly described pathogenic role of dysregulated NET formation in syndromes of pathologic inflammation (28, 29).

## Ethics Statement

Institutional Review Board – University of Utah School of Medicine: IRB Protocol #056286. Pediatric participants were recruited by Meghann P. McManus, D.O., Michael A. Pulsipher, M.D., and Michael Boyer, M.D. (Co-Investigators and Pediatric Bone Marrow Transplant Physicians) as they met with patients to coordinate their bone marrow transplant procedure. For adult study participants, patients were recruited by Michael A. Pulsipher, M.D., Michael Boyer, M.D., Thai Cao, M.D., and Schickwann Tsai, M.D. (Co-Investigators and Adult Bone Marrow Transplant Physicians). Informed consent was sought at the time when the patients were consented for their bone marrow transplant procedure. The study information and procedures were discussed in detail with each patient and their family as applicable (pediatric patients). A copy of the study associated with Consent and Authorization Document (Adult) or Parental Permission and Authorization Document (Pediatric) was given to the individual patient or family to read. Time was provided for the individual and family to consider the issues and discuss their response privately. No attempts at coercion or use of undue influence were made. The patients and families were clearly told that refusal of consent for this study would not in any way change the attitude or care of the clinical staff to the patient.

## Author Contributions

JG performed, directed, and interpreted experiments, and wrote significant portions of the manuscript. MC performed experiments, provided key experimental approaches, and interpreted results. MM recruited study participants, collected participant blood samples, and assisted with collection and characterization of participant clinical demographic and treatment data. MP recruited study participants, interpreted experimental results, provided key clinical expertise in the area of BMT, and edited portions of the manuscript. JS participated in study conceptualization, recruitment of study participants, edited portions of the manuscript, and provided key clinical insight into human cancer diagnosis and treatment. CY provided overall direction and conceptualization to the project, provided expertise regarding neutrophil biology and NET formation, reviewed and analyzed all experiments, wrote sections of the manuscript, and edited all portions of the manuscript.

## Conflict of Interest Statement

The authors declare that the research was conducted in the absence of any commercial or financial relationships that could be construed as a potential conflict of interest.
